# Identification of Novel Mutations in the N Gene of SARS-CoV-2 That Adversely Affect the Detection of the Virus by Reverse Transcription-Quantitative PCR

**DOI:** 10.1128/spectrum.00545-21

**Published:** 2021-08-25

**Authors:** Rashedul Hasan, Mohammad Enayet Hossain, Mojnu Miah, Md Mahmudul Hasan, Mustafizur Rahman, Mohammed Ziaur Rahman

**Affiliations:** a International Centre for Diarrhoeal Disease Research, Bangladesh, Dhaka, Bangladesh; Houston Methodist Hospital

**Keywords:** SARS-CoV-2, COVID-19, RT-qPCR, N gene, mutation

## Abstract

Accurate and timely diagnosis of SARS-CoV-2 is a critical step toward controlling the viral spread, since it facilitates the identification and isolation of infected individuals. Mutations in the primer-/probe-binding sites may lead to false-negative results.

## LETTER

COVID-19 originated in late 2019 in Wuhan, China, engendering a pandemic. As of 4 June 2021, there had been more than 173 million confirmed cases and more than 3.7 million deaths reported globally. Laboratory diagnosis of SARS-CoV-2 is the mainstay for alleviating viral spread, since it facilitates the identification and isolation of infected individuals. Since reverse transcription-quantitative PCR (RT-qPCR) oligonucleotides, primers, and probes bind to a diminutive, ∼20- to 25-bp region, mutations in this region can potentiate impaired primer and/or probe binding and subsequent inefficient PCR amplification, thereby spawning false negatives. Given the high transmissibility of SARS-CoV-2 (1), such false negatives could have affected the containment of COVID-19 outbreaks. Genetic evolution is a widespread and natural phenomenon for RNA viruses. SARS-CoV-2 is an RNA virus. The nature of its evolution contributes to the emergence of new variants and its demonstrated diagnostic nonuniformity, especially when conducting RT-qPCR using different primers and probes (2).

Here, we report the detection of novel mutations in the N gene of SARS-CoV-2 corresponding to the China CDC N RT-qPCR assay and the determination of the frequency in sequences submitted from Bangladesh.

**FIG 1:**
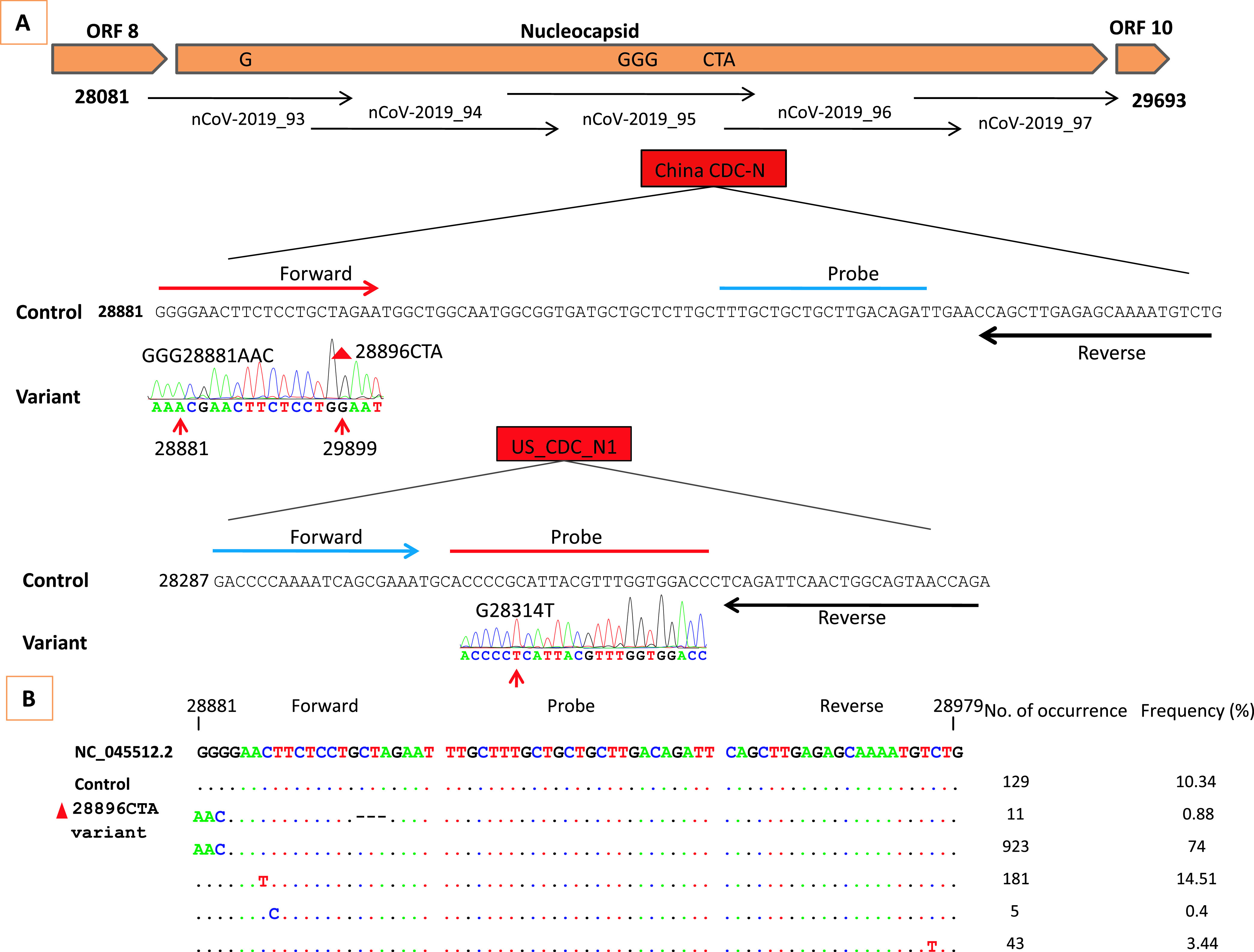
(A) Graphic representation of mutations in the N gene of a SARS-CoV-2 virus and strategy to unveil the suspected mutations linked to detection failure in the China CDC N RT-qPCR assay. The arrows and numbers correspond to the fragments of the ARTIC primer sets for N gene amplification. (B) Frequency of the N gene mutations in the primer-binding regions detected in the study in 1,245 viral genome sequences from Bangladesh. The dots indicate nucleotides identical to those of the primers and probes.

The International Centre for Diarrhoeal Disease Research, Bangladesh (icddr,b), in association with the government of Bangladesh, has been actively testing for SARS-CoV-2 since March 2020 as part of the countrywide COVID-19 laboratory network. We have been using the World Health Organization (WHO)-recommended RT-qPCR protocol for the detection of the ORF1ab and N genes of SARS-CoV-2 (3). Occasionally, we have observed two issues during the RT-qPCR: (i) positive results with cycle threshold (C_T_) values of 21.66 and 17.48 for the ORF1ab region, but no curve for the N genes, and (ii) a higher C_T_ value for the N gene compared to the ORF1ab region (31.55 versus 15.10, respectively). The target samples were retested using the US CDC novel coronavirus (nCoV) N1 RT-qPCR assay (3), which yielded positive results for all the targets. Because of the failure of the current assay to amplify the N gene, mutations in the primer- and/or probe-binding sites on the SARS-CoV-2 N gene were suspected. To identify the possible mutations responsible for the PCR failure, we sequenced the entire N gene of two controls and three selected samples using Sanger sequencing. cDNA was prepared using the high-capacity cDNA reverse transcription kit (Thermo Fisher Scientific, CA, USA) as per the kit instructions. The N gene was amplified using the ARTIC primer sets (4) with GoTaq G2 Hot Start *Taq* polymerase (Promega, WI, USA). Nucleotide sequencing was performed in an automated ABI 3500xL genetic analyzer (Applied Biosystems, Foster City, CA) using the BigDye Terminator v3.1 cycle sequencing kit (Applied Biosystems). The sequenced data were inspected by BLAST search, contigs were generated using the SeqMan tool (DNASTAR, Inc.), and multiple sequence alignment was performed using the BioEdit sequence alignment editor. Triple nucleotide substitutions (GGG28881AAC) at the 5′ end and a triple nucleotide deletion (28896CTAdel) at the 3′ end of the forward primer-binding site (3) were detected in the suspected samples (Fig. 1A). Interestingly, one nucleotide substitution (G28314T) was also observed in the probe-binding region for the U.S. CDC nCoV N1 RT-qPCR assay, although the mismatch did not impair detection in the confirmatory test (Fig. 1A).

To estimate the frequency of both the substitutions and the deletion among Bangladeshi strains, we retrieved all complete SARS-CoV-2 sequences (1,247 sequences) from the GISAID EpiCoV database (28 April 2021). Nucleotide mismatches in the Bangladeshi sequences are described in Fig. 1B. In particular, the GGG28881AAC substitution and the 28896CTA deletion were simultaneously detected in 11 sequences (0.88%). According to a local BLASTN search, 40 isolates carry both the 28881AAC variant and the 28896CTA deletion. Based on the GISAID EpiCoV database, 48.8% (649,115/1,329,560) sequences contain the 28881AAC variant (5). Approximately 0.18% (*n* = 3,803) of the isolate sequences in GISAID contain the target 28896CTA deletion, and 1.54% of the genomes contain at least one mutation in the last pentanucleotides of the 3′ primer region for the China CDC N RT-qPCR assay as reported by the GISAID EpiCoV database (5). Isolates containing both the 28881AAC variant and the 28896CTA deletion, which stopped detection of the N gene using the China CDC N RT-qPCR assay, originated from the United States, Australia, England, and Bangladesh. According to the U.S. CDC nCoV N1 RT-qPCR assay, the probe region contains about 10,105 single-nucleotide polymorphisms (SNPs). The G28314T substitution is carried by 39 and 25 sequences according to GISAID and BLASTN searches, respectively.

RNA viruses such as SARS-CoV-2 are prone to mutations associated with virulence and evolvability, traits considered beneficial for viruses. Mutations in the primer- and probe-binding regions may lead to false-negative diagnoses for SARS-CoV-2. Previous studies have claimed that a single mutation (C29200T or C29200A) in the probe-binding region of an isolate was associated with failure of the virus detection when using the U.S. CDC nCoV N1 RT-qPCR assay (6, 7).

Our data show that SARS-CoV-2 variants carrying mutations in the primer-/probe-binding regions that impair RT-qPCR detection may have emerged and spread in the community. Our study suggests that mutations in the SARS-CoV-2 N gene RT-qPCR primer-binding sites might cause issues related to sensitivity and potentially underdiagnosis if we rely on an assay with only a single target. Mutations in the primer-/probe-binding regions of both targets could potentially result in false-negative detection. According to the GISAID update on common primer checks, the primer-/probe-binding regions for the targets of all WHO in-house SARS-CoV-2 diagnostic assays carried mutations, suggesting the need for continuous monitoring for mutations, especially when one of the RT-qPCR assays fails to detect SARS-CoV-2 (5). This study also highlights the importance of the use of RT-qPCR assays with at least two targets for SARS-CoV-2 detection. If symptomatic COVID-19 patients test negative using one assay, our findings strongly support retesting with another assay to avoid false-negative results.

### Data availability.

The sequence data have been submitted to GenBank (accession no. MZ379157 and MZ379158) and GISAID (accession no. EPI_ISL_1715168).

### Ethical statement.

This study has been approved by the research review board of icddr,b (protocol no. PR-20102).
